# Institutional Reductions in Opioid Prescribing Following Hip Arthroscopy Do Not Change Patient Satisfaction Scores

**DOI:** 10.1016/j.asmr.2020.10.013

**Published:** 2021-02-25

**Authors:** David A. Bloom, Amit K. Manjunath, Charles Wang, Alexander J. Egol, Robert J. Meislin, Thomas Youm, Guillem Gonzalez-Lomas

**Affiliations:** NYU Langone Health, New York, New York, U.S.A.

## Abstract

**Purpose:**

To investigate what effect decreased opioid prescribing following hip arthroscopy had on Press–Ganey satisfaction survey scores.

**Methods:**

A retrospective review of prospectively collected data was conducted on patients who underwent primary hip arthroscopy for femoroacetabular impingement between October 2014 and October 2019. Inclusion criteria consisted of complete Press–Ganey survey information, no history of trauma, fracture, connective tissue disease, developmental hip dysplasia, autoimmune disease, or previous hip arthroscopy. Groups were separated based on date of surgery relative to implementation of an institutional opioid reduction policy that occurred in October 2018. Prescriptions were converted to milligram morphine equivalents (MME) for direct comparison between different opioids.

**Results:**

A total of 113 patients met inclusion criteria, 88 preprotocol and 25 postprotocol. There were no statistically significant differences between groups with respect to patient demographics or intraoperative pathologies (*P* > .05). Average opioid prescription dropped from 249.6 ± 152 MME (equivalent to 33.3 tablets of oxycodone 5 mg) preprotocol to 108.6 ± 84.7 MME (equivalent to 14.5 tablets of oxycodone 5 mg) postprotocol; *P* = .0002. There were no statistically significant differences in Press–Ganey survey scores between pre- and postprotocol groups (*P* > .05).

**Conclusions:**

A reduction in opioids prescribed after a hip arthroscopy is not associated with any statistically significant difference in patient satisfaction with pain management, as measured by the Press–Ganey survey.

**Level of Evidence:**

Level III, retrospective comparative study.

The opioid epidemic was responsible for more than 46,000 deaths in the United States in 2018.^1^ While awareness and advocacy have increased tremendously, there remain many areas for improvement. Orthopaedic surgeons are the third-greatest prescribers of opioids among all specialties and therefore play a significant role in both the cause and the solution to this epidemic.^1^ Opioid-based analgesia routinely has been prescribed to manage acute pain after orthopaedic procedures.^2^ Overprescription has been linked with physician desire to properly manage patient expectations, along with fear of potential consequences associated with undertreating pain.^3^

Existing literature has suggested that overprescribing is linked, at least in part, to a physician desire to mitigate poor patient satisfaction ratings, despite providing otherwise positive health care.^3^, ^4^, ^5^ The Press–Ganey (PG) survey is a commonly administered patient satisfaction tool that measures patient perception of care, using similar metrics as the Hospital Consumer Assessment of Healthcare Providers and Systems survey, which is used as a reimbursement metric by the Value-Based Purchasing program.^6^ These programs place great emphasis on the patient’s perception of care, and pain control remains a major metric in this paradigm.^3^^,^^4^

Previous research by Kohring et al.^7^ demonstrated no correlation between PG scores and patient-reported outcome scores in patients undergoing total hip arthroplasty. Recently, Bloom et al.^8^ demonstrated that PG pain control survey scores did not decrease when patients were given fewer opioids following total shoulder arthroplasty.

The purpose of this study was to investigate what effect decreased opioid prescribing following hip arthroscopy had on PG satisfaction survey scores. We hypothesize that patient satisfaction would not change, despite patients being prescribed less opioid pain medication.

## Methods

### Study Design and Participants

This single-center, institutional review board–approved study involved the query of this institution’s electronic medical record for all patients who underwent primary hip arthroscopy for femoroacetabular impingement between October 1, 2014 and October 1, 2019.

Inclusion criteria for this study were as follows: patients aged ≥18 years old on day of surgery who underwent primary hip arthroscopy for femoroacetabular impingement with an associated PG survey were included. Patients were excluded if they were outside the inclusion age limit, revision cases, had a connective tissue disorder, or whose hip problems were related to traumatic fracture. Patients were similarly excluded if they were enrolled in any research studies involving pain management, opioid consumption, anesthesia, or rheumatology. In addition, patients were excluded if data to be analyzed were missing. Following preliminary chart review, demographic information (age, sex, BMI, date of surgery, previous opioid use, and smoking status) was recorded. In addition, any information regarding previous surgery on an upper extremity was recorded.

### Surgical Technique

All patients were placed under general anesthesia before the start of the procedure. Surgery was performed by 1 of 3 authors (R.J.M., T.Y., G.G.L.). All patients underwent hip arthroscopy in traction, in the supine position. Typically, an anterolateral portal was made with fluoroscopy before a second, mid-anterior portal was made under direct visualization. Then, an interportal capsulotomy was performed to allow access to the central compartment for chondrolabral debridement or repair.

When needed, an anterolateral accessory portal was made distally to assist in anchor placement for the purposes of labral repair. Cam-type lesion osteochondroplasty or acetabular rim trimming was performed if needed. Rarely, loose body removal or subspine impingement resection was performed, and only if needed. Chondral debridement was performed with electrocautery or shaver. Microfracture was not performed in any of these patients. At the conclusion of the procedure, capsular repair was conducted.

### Perioperative Care

Patients underwent hip arthroscopy in an outpatient setting. They were given a hip abduction brace so as to limit both hyperextension and external rotation and restricted to flat-foot weight-bearing on their operative extremity with crutch-support for the first 3 to 4 weeks postoperatively. Patients were discharged with 12 tablets of 500 mg cephalexin, taken every 6 hours for the first 3 days for infection prophylaxis. They were also given 7 tablets of 81 mg aspirin for venous thromboembolism prophylaxis, and they were given a 14-day supply of either Celebrex (200 mg daily) or Naproxen (500 mg daily) for heterotopic ossification prophylaxis. Patients were prescribed physical therapy with the goal of return to sports at 6 months after surgery.

An opioid-sparse institutional protocol for perioperative pain management was enacted in October 2018, and before that, opioid prescriptions were surgeon-specific. In October 2018, a formal protocol was enacted, which included giving patients up to 20 tablets of oxycodone/acetaminophen 5 mg/325 mg.

### PG Survey

Patients responded to surveys, administered postoperatively by PG, via mail or e-mail. If surveys were not returned within 1 month, a second questionnaire was sent. These surveys consisted of a modified version of the Hospital Consumer Assessment of Healthcare Provider and Systems questionnaire survey. This survey is a multi-item, patient-focused instrument administered to evaluate patient satisfaction with their perioperative experience, which includes pain management.

There were 4 survey questions of interest: (1) “degree to which your pain was controlled”; (2) “Nurses’ concern for your comfort after the procedure”; (3) “Explanation the physician gave you about what the surgery or procedure would be like”; and (4) “Information the physician provided you about what was done during your procedure.” These questions were scored in a Likert-type fashion, from 1 (lowest) to 5 (highest).

### Variables of Interest

The primary outcomes of interest in this study were the following: (1) Discharge opioid prescription quantity and (2) patient satisfaction with pain control, as determined by a Likert-type scaled response to the survey question “rate the degree to which your pain was controlled (1-5).” PG data were obtained from our institution’s PG representative. Postoperative opioid prescriptions were converted from milligrams or milliliters to milligram morphine equivalents (MME) for direct comparison between different opioids and were obtained from direct institutional electronic medical record chart review.

Demographic data were analyzed and recorded from additional chart review, intraoperative pathology was similarly recorded from operative notes, and several other survey questions were analyzed for statistical significance. Patients were divided into 2 chronological groups, before and after protocol commencement, for comparison. These intervals included October 31, 2014 (date of first surgery) to October 1, 2018 (date of implementation of institutional perioperative opioid policy), and October 2, 2018, to September 6, 2019. To add further validity to our data and existing body of literature on the subject, we duplicated the methods of Daniels et al.^4^ and also categorized these numerical satisfaction responses into 2 groups, with “4”s and “5”s (highest satisfaction) being considered “satisfied” and “1s, 2s, and 3s” being considered “dissatisfied.”

For the determination of clinical significance, the minimum detectable change (MDC) was calculated. The MDC value, the smallest difference in 2 scores that is considered larger than measurement error, is considered the most conservative estimate of minimal clinically important difference.^9^ Accordingly, differences less than MDC may be considered clinically insignificant, and values greater than the calculated MDC may have clinical significance. This study included 5 years of data (4 preprotocol and 1 postprotocol); we calculated that we would need at least 88 patients preprotocol and 22 patients postprotocol to demonstrate significance between groups based on the smallest calculated MDC (MDC = 0.345) for responses to the “satisfaction with pain control” question, with an associated α of 0.05 and power (1 – β) = 0.80.

### Statistical Analysis

All continuous data were initially tested for normality via a D’Agostino-Pearson test. Direct group comparisons for pain control and MME were performed with the nonparametric Mann–Whitney test, as the groups failed a D’Agostino-Pearson test for normality. In addition, χ^2^ analysis was used for dichotomous outcome analysis. χ^2^ analysis also was used to when Likert-type satisfaction data were reanalyzed as a dichotomous outcome. All statistical analysis was performed using GraphPad Prism 8.4 (GraphPad Software, La Jolla, CA). Statistical significance was set at *P* < .05.

MDC was calculated with the accepted formula of standard error of measure × 1.96 × √2 where the standard error of measure is calculated as standard deviation × √(1 – r), where “r” is the test–retest reliability coefficient.^10^ This study used a previously determined value of “r,” 0.92, the most conservative calculation of Fulton et al.^11^

## Results

### Patient Demographics

A total of 115 patients were identified who underwent hip arthroscopy and had an associated, complete postoperative PG survey. Of those, 2 were excluded based on demographic information, medical history, or surgical indication. Average age of the overall cohort was 40.33 ± 13.1 years, with an average BMI of 25.33 ± 4.7 kg/m^2^. Average opioids prescribed at discharge were 218.4 ± 151 MME over the study period. Average patient satisfaction with pain control was 4.77 ± 0.60 of a possible 5.

Table 1 demonstrates these demographic data before and after the implementation of this institution’s perioperative opioid-sparse pain protocol. There were no statistically significant differences (*P* > .05) between pre- and postimplementation of the formalized pain protocol.

**Table 1:** Patient Demographics

Variable	Before Protocol (N = 88)	After Protocol (N = 25)	*P* Value
Age, y	41.40 ± 12.7 [38.72-44.08]	36.56 ± 14.0 [30.79-42.33]	.10
BMI (kg/m^2^)	25.47 ± 4.9 [24.40-26.54]	24.83 ± 4.3 [22.95-26.71]	.62
Sex, female	72 (81.8%)	20 (80.0%)	.78
Smoking status, yes	20 (22.7%)	6 (20.0%)	.99
Previous opioid use, yes	38 (43.2%)	10 (40.0%)	.82
Opioid-naïve, yes	81 (92.1%)	24 (96.0%)	.68
Refill, yes	9 (10.3%)	3 (12.0%)	.73

NOTE. Data are shown as mean ± standard deviation, no. patients (%), or [95% confidence interval].

Table 2 demonstrates that there were no statistically significant differences between groups with respect to intraoperative pathology, which included chondral delamination, labral tear, cam-type lesions, and pincer-type lesions (*P* > .05 for all). In addition, 89.8% (79) of patients preprotocol underwent labral repair, compared with 88.0% (22) postprotocol (*P* = .726). 12.5% (11) of patients preprotocol underwent labral debridement, compared with 16.0% (4) postprotocol (*P* = .739).

**Table 2:** Patient Demographics

Variable	Before Protocol (N = 88)	After Protocol (N = 25)	*P* Value
Chondral delamination	87 (98.9%)	23 (92.0%)	.12
Labral tear	87 (98.9%)	25 (100%)	>.99
Cam-type lesion	68 (77.3%)	23 (92.0%)	.15
Pincer-type lesion	77 (87.5%)	23 (92.0%)	.73

NOTE. Data are shown as no. patients (%).

### Pre- Versus Postprotocol Opioid Prescribing

**Fig 1:**
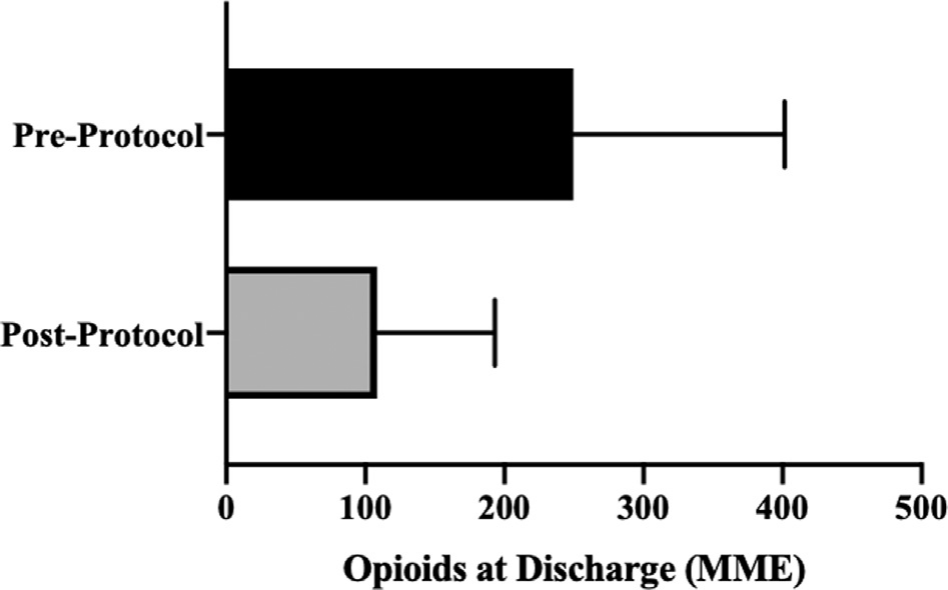
Comparison of preprotocol and postprotocol opioid prescription in MME. (MME, milligram morphine equivalents.)

As seen by Fig 1, there was a statistically significant decrease in narcotic prescribing following initiation of this institution’s novel pain management protocol. Mean discharge opioids for the preprotocol group was 249.6 ± 152 MME (equivalent to 33.3 tablets of oxycodone 5 mg), and postprotocol it was 108.6 ± 84.7 MME (equivalent to 14.5 tablets of oxycodone 5 mg); *P* = .0002, power (1 – β) = 0.99. This was equivalent to a 56.5% reduction in average postoperative opioid prescription following hip arthroscopy.

### PG Scores Versus Protocol Implementation

When PG scores were separated into preprotocol and postprotocol cohorts and analyzed for statistical significance via Mann–Whitney test, there were no statistically significant differences in scores between groups (*P* > .05). The results of this analysis can be seen in Table 3.

**Table 3:** Press–Ganey Scores Pre- and Postprotocol Implementation

Variable	Before Protocol (N = 88)	After Protocol (N = 25)	*P* Value
Degree to which your pain was controlled (1-5)	4.78 ± 0.64 [4.65-4.92]	4.76 ± 0.44 [4.58-4.94]	.36
Nurses’ concern for your comfort after the procedure (1-5)	4.73 ± 0.69 [4.58-4.87]	4.68 ± 0.69 [4.40-4.97]	.81
Explanation the physician gave you about what the surgery or procedure would be like (1-5)	4.86 ± 0.38 [4.78-4.94]	4.84 ± 0.37 [4.67-4.99]	.74
Information the physician provided about what was done during your surgery or procedure (1-5)	4.66 ± 0.87 [4.48-4.84]	4.92 ± 0.28 [4.81-5.00]	.23

Data are shown as mean ± standard deviation or [95% confidence interval].

When these PG questions were converted to dichotomous variables for further comparison per the methods of Daniels et al.,^4^ there were no statistically significant differences between groups. Of the preprotocol group, 84 (95.5%) answered favorable to the question “degree to which your pain was controlled,” compared with 25 (100%) from the postprotocol group, *P* = .57. None of the other variables were found to demonstrate any statistically significant differences and are seen in Table 4.

**Table 4:** Press–Ganey Scores Pre- and Postprotocol Implementation—Dichotomous Satisfaction

Variable	Before Protocol (N = 88)	After Protocol (N = 25)	*P* Value
Degree to which your pain was controlled (yes)	84 (95.5%)	25 (100%)	.57
Nurses’ concern for your comfort after the procedure (yes)	83 (94.3%)	22 (88.0%)	.37
Explanation the physician gave you about what the surgery or procedure would be like (yes)	87 (98.9%)	24 (96.0%)	.40
Information the physician provided about what was done during your surgery or procedure (yes)	79 (89.8%)	25 (100%)	.20

Data are shown as no. patients satisfied (%)

## Discussion

The most important finding of this study was that despite opioid-based analgesia being a mainstay of treatment for immediate postoperative pain in patients undergoing hip arthroscopy, a reduction in postoperative prescribing did not result in a statistically significant change in patient satisfaction with pain control. Orthopaedic surgeons have a responsibility to reduce our footprint on the opioid epidemic, all while maintaining high patient satisfaction, especially by maintaining low levels of postoperative pain. In an effort to mitigate excessive opioid prescribing, our institution implemented opioid prescribing guidelines that significantly reduced the number of pills patients received postoperatively. We found no association between the reduced prescription of opioid medication at discharge and a change in postoperative patient satisfaction with pain control, as measured by PG survey responses.

The 5-year study period (2014-2019) served as an ideal period because it allowed comparison before and after the implementation of institutionally imposed postoperative opioid prescribing guidelines. During the pre-protocol period, both federal and state regulations were instituted to reduce opioid prescribing. The New York State government passed legislation in 2016, which prevented providers from prescribing more than a 7-day supply of an opioid medication for acute pain. The United States government passed the 21st Century Cure Act in 2017, officially naming the ongoing opioid epidemic a national public health emergency.^12^ In the same year, the Center for Medicare and Medicaid Services issued formal guidelines aimed at restricting the amount of opioids that Medicare beneficiaries could receive.^12^

This institution’s protocol appears to have been very effective, as the mean volume of opioids prescribed significantly decreased after implementation. It is also important to recall the duration of study period, which suggests that these decreased prescribing patterns are durable, having persisted for at least 1 year.

Previous research has suggested that one obstacle to the widespread implementation of decreased opioid-prescribing protocols is concern that narcotic reduction may lead to lower patient satisfaction scores.^13^ These satisfaction metrics are important for health care center valuations but also have the ability to influence the care-providing, itself. Merriman et al.^3^ recently published the results of a survey sent to emergency medicine physicians and reported that 50% of physicians believe patients are more satisfied when given opioid medications in the emergency department or as a discharge prescription. Perhaps more significantly, roughly 30% of those surveyed believed that patient satisfaction survey scores are superior when given opioid medications (either in emergency department as discharge prescription).^3^ These physicians responded saying that patient expectations (95%) and peer-review journals (100%) were the 2 leading sources of pressure to prescribe more opioid medications at discharge.^3^

Value-based purchasing initiatives incorporate patient perception of care into a reimbursement algorithm that then determines provider and hospital level compensation. The implicit relationship between patient pain, functional outcome scores, and patient satisfaction surveys has been previously researched. Rane et al.^14^ sought to delineate the relationship between patient-reported pain with patient satisfaction and discovered that with every 10-point increase in Patient-Reported Outcomes Measurement Information System (PROMIS) Pain Interference, there was a corresponding 17% decrease in the odds of overall satisfaction [21]. In this study, we demonstrated consistently high patient satisfaction despite marked reduction in postoperative opioid prescribing, which is in line with the literature for other orthopaedic ambulatory procedures. Etcheson et al.^15^ demonstrated that PG patient satisfaction scores are not influenced by postoperative opioid use after total hip arthroplasty and total knee replacement. Other studies have demonstrated that administration of narcotics does not correlate with greater patient satisfaction scores.^16^^,^^17^ This is a critical conclusion that highlights the common misconception that opioids are essential for maintaining patient satisfaction through better pain control.

This is of substantial clinical significance to practicing hip arthroscopists for at least 2 reasons. First, previous work by Westermann et al.^18^ has demonstrated that nearly half (45%) of patients undergoing hip arthroscopy have a recent history (within 3 months) of opioid use. This suggests that most, if not all, providers will encounter these patients with regularity. Second, the work of Westermann et al.^18^ has implications for clinical research—as they demonstrated that patients classified as “current users” had statistically significant lower baseline function, as measured by several patient-reported outcome scores.

The results of our study demonstrate a 56.5% reduction in average postoperative opioid prescribing, somewhat larger than the 36.0% reduction associated with a study by Stepan et al.^19^ Their institutional policy was formed as the result of a combination of examining available literature, available prescribing guidelines, and a surgeon-based poll on prescribing patterns. After establishing an institution-wide narcotic-prescribing education program, Stepan et al.^19^ discovered significant reductions in opioid prescribing behaviors across multiple ambulatory procedures including knee arthroscopy, hip arthroscopy, shoulder arthroscopy, carpal tunnel releases, and distal radius fractures. This change in prescribing behavior was noted to potentially result in a decrease of almost 30,000 fewer opioid pills prescribed per year. When examining the results for hip arthroscopy, Stepan et al.^19^ demonstrated a preprotocol average of 334.0 ± 110 MME (equivalent to 44.5 ± 14.7 oxycodone 5 mg) and a postprotocol average of 213.9 ± 73.8 MME (28.5 ± 9.7 oxycodone 5 mg). This finding has special relevance when considering the results reported by Anciano Granadillo et al.,^20^ who found that more than 25% of patients undergoing hip arthroscopy were found to continue receiving opioid prescriptions more than 3 months postoperatively. Importantly, those receiving chronic prescriptions were noted to be at greater risk for revision hip arthroscopy and conversion to total hip arthroplasty.^20^

It is important to note that many studies are able to demonstrate a decrease in opioid use through a growing emphasis and implementation of multimodal pain management techniques as well as perioperative interventional pain management. In a systemic review published in 2018, Shin et al. analyzed published randomized controlled trials and comparative studies on pain control after hip arthroscopy.^21^ Femoral nerve blocks, lumbar plexus blocks, local anesthetic infiltration at the surgical site, and periacetabular injections were noted to decrease opioid consumption postoperatively.^21^ In addition, as we become more familiar with the multitude of biochemical pain pathways, different classes of pain medications have become areas of interest due to their possible synergistic pain-relief effects. In a 2017 prospective randomized placebo-controlled study, Kahlenberg et al.^22^ reported statistically significant lower pain scores and discharge time in hip arthroscopy patients who received preoperative celecoxib.

Thus, it stands to reason that there are viable alternatives to the opioid-predominant pain regimens, and that reductions in opioid-prescribing have no statistically significant impact on increasingly important patient satisfaction surveys. In addition, provider-focused opioid education may be a viable option to further reduce overall opioid prescribing at the institutional level and should be pursued. In addition, investigation regarding a multimodal and multidisciplinary approach to pain management after hip arthroscopy may potentially curtail opioid consumption even further, without jeopardizing patient satisfaction with pain control.

### Limitations

This study is not without limitations. First is its retrospective nature. Second, our results are at risk for responder bias. Patients did not keep a diary nor record the actual consumption of narcotics. Next, there are possible ceiling effects of the survey. It is unclear how sensitive the survey would be to small changes in only the one domain, and how that could affect the results and analysis. Finally, we did not control for confounding variables (most commonly patient demographic, age, etc.) that are known to influence PG scores.

## Conclusions

A reduction in opioids prescribed after a hip arthroscopy is not associated with any statistically significant difference in patient satisfaction with pain management, as measured by the PG survey.

## Supplementary Data

ICMJE author disclosure forms
